# Influenza A Strain-Dependent Pathogenesis in Fatal H1N1 and H5N1 Subtype Infections of Mice

**DOI:** 10.3201/eid1604.091061

**Published:** 2010-04

**Authors:** Mutien-Marie Garigliany, Adélite Habyarimana, Bénédicte Lambrecht, Els Van de Paar, Anne Cornet, Thierry van den Berg, Daniel Desmecht

**Affiliations:** University of Liège, Liège, Belgium (M.-M. Garigliany, E. Van de Paar, A. Cornet, D. Desmecht); Veterinary Agrochemical Center, Brussels, Belgium (A. Habyarimana, B. Lambrecht, T. van den Berg).

**Keywords:** Influenza, viruses, lung, acute respiratory distress syndrome, ARDS, research

## Abstract

To determine if fatal infections caused by different highly virulent influenza A viruses share the same pathogenesis, we compared 2 different influenza A virus subtypes, H1N1 and H5N1. The subtypes, which had shown no pathogenicity in laboratory mice, were forced to evolve by serial passaging. Although both adapted viruses evoked diffuse alveolar damage and showed a similar 50% mouse lethal dose and the same peak lung concentration, each had a distinct pathologic signature and caused a different course of acute respiratory distress syndrome. In the absence of any virus labeling, a histologist could readily distinguish infections caused by these 2 viruses. The different histologic features described in this study here refute the hypothesis of a single, universal cytokine storm underlying all fatal influenza diseases. Research is thus crucially needed to identify sets of virulence markers and to examine whether treatment should be tailored to the influenza virus pathotype.

## Abstract 2

Future treatments may involve customizing treatment to the virus pathotype.

According to the World Health Organization, influenza annually infects 5%–15% of the global population, causing 3–5 million cases of severe illness and ≈500,000 reported deaths. The persistence of influenza A virus (H5N1) in poultry populations over the past 6 years and the ability of those viruses to cause fatal infections in humans, along with the recent pandemic (H1N1) 2009 outbreaks, have raised fears of a renewed catastrophic influenza outbreak comparable to that of 1918, which caused death in 0.2%–8% of those infected in various countries and ≈50 million deaths worldwide (*1*). Standard influenza symptoms include fever, cough, headache, sore throat, and dehydration, with some reports of diarrhea, vomiting, and bleeding from the mouth or throat. In benign cases, not all of these symptoms are exhibited. In severe cases, additional signs typical of either secondary bacterial pneumonia or acute respiratory distress syndrome (ARDS) occur. Notably, these 2 manifestations are those that cause death in patients with influenza, whether seasonal or pandemic or caused by the 1918 subtype H1N1 strain or by recent subtype H5N1 strains.

The catastrophic lethality of the 1918 pandemic makes it paramount that we understand the disease pathogenesis of both severe forms of influenza. Because most secondary bacterial pneumonias can be controlled with antimicrobial agents, prevention and treatment of influenza-associated ARDS are the major medical challenges that must be addressed to reduce the influenza-related death rate. This requires more knowledge about the pathogenesis of ARDS. Alterations in human and mouse lungs have been described for fatal virus infections with pandemic virus strains (subtypes H1N1, H2N2, and H3N2 strains of 1918, 1957, and 1968, respectively) or subtype H5N1 strains. They are all characterized by similar lung dysfunctions and lesions (*2**,**3*). The lung becomes flooded as its alveolocapillary membranes leak, and the alveoli fill with body fluids. Consequently, the exchange of carbon dioxide and oxygen is reduced, and fatal acute lung failure ensues. The histologic findings depend on the stage of the disease. Edema, epithelial necrosis, fibrin, and hyaline membranes are found during the early exudative phase, and fibroblast and type II cell hyperplasia are found during the proliferative phase. This array of morphologic alterations is known as diffuse alveolar damage. Moreover, mice infected with the 1918 influenza virus or with a recent subtype H5N1 human isolate also show considerable similarities in overall lung cellularity, composition of lung immune cell subpopulation, and cellular immune temporal dynamics (*4*). On the basis of these mostly retrospective studies, the pathogenesis of influenza-associated ARDS is widely viewed as being the same whatever the infecting strain.

In this study, we closely monitored ARDS in mice, caused by inoculation of identical doses of 2 different influenza strains rendered highly pathogenic toward mice by adaptation. The 2 strains elicited dramatically different disease courses and histopathologic signatures, although both strains caused death in 100% of those infected, evoked the expected diffuse alveolar damage, and led to comparable virus titers in the lungs. The pathogenesis underlying influenza-associated fatal ARDS thus depended on the infecting strain.

## Materials and Methods

### Animals

Eight-week-old female FVB/J mice weighing 20–25 g were obtained from Charles River Laboratories (L’Arbresle, France). Challenge studies were conducted under BioSafety Level 3 laboratory conditions and in facilities accredited by the Belgian Council for Laboratory Animal Science, under the guidance of the Institutional Animal Care and Use Committees of the Veterinary Agrochemical Research Center and University of Liège. The mice were housed in microisolator cages ventilated under negative pressure with HEPA-filtered air. The light/dark cycle was 12/12 h, and the animals were allowed free access to food and water. Before each inoculation or euthanasia procedure, the animals were anesthetized by intraperitoneal injection of a mixture of ketamine (50 mg/kg) and xylazine (30 mg/kg).

### Viruses

Two influenza A virus strain subtypes that had low pathogenicity for laboratory mice were used in this study: a clade 1 avian influenza virus (H5N1) (A/crested_eagle/Belgium/1/2004), and a porcine influenza virus (H1N1) (A/swine/Iowa/4/76). Both viruses were first propagated in the allantoic cavity of 10-day-old embryonating hen eggs and then adapted to the mice by lung-to-lung passaging. At each passage, a set of mice were inoculated intranasally with 50 µL of either allantoic fluid or lung homogenate containing influenza A virus. At 5 days postinoculation (dpi), the mice were killed humanely by an overdose of pentobarbital, followed by exsanguination. The lungs were combined and homogenized in phosphate-buffered saline (PBS)–penicillin-streptomycin, the homogenates were centrifuged at 3,000 g for 10 min, and the supernatant was used for the next passage. The process was stopped when the mice showed a substantial loss of bodyweight on 4 dpi. This occurred after 5 (H5N1) or 31 (H1N1) passages. Lung homogenates from the last passage were homogenized and divided into aliquots for direct use in pathotyping studies, and their titers were determined by standard plaque (subtype H1N1) or median tissue culture infective dose assays (H5N1). Serial dilutions of each adapted virus stock were then injected into FVB/J mice, and the 50% mouse lethal dose (MLD_50_) was calculated according to the method of Reed and Muench (*5*).

### Pathotyping Studies

For assessment of virus-induced pathogenicity, 2 series of mice were inoculated intranasally with 10 MLD_50_ of virus by instillation of 50 µL of diluted stock. Mice were monitored daily for changes in bodyweight to assess virus-induced illness. At selected intervals, 5 (virus titration or histopathology) or 10 (virus titration + dry/wet weight ratio) mice were given an overdose of sodium pentobarbital and exsanguinated by cutting the brachial artery. Lungs and pieces of heart, liver, spleen, pancreas, kidney, brain, and adipose tissue from 5 mice were fixed in 4% neutral-buffered, ice-cold paraformaldehyde, routinely processed, and embedded in paraffin for histopathologic evaluation. Five-micrometer sections were stained with hematoxylin and eosin (HE) or periodic acid–Schiff (PAS) for lesion detection. For virus detection, sections were stained by a streptavidin-biotin complex immunoperoxidase method. An in-house immunoglobulin (Ig) G–purified polyclonal rabbit antiserum raised against recombinant influenza virus nucleoprotein was used as the source of primary antibodies, and horseradish peroxidase (HRP)–conjugated anti–rabbit IgGs (Dako, Glostrup, Denmark) were used as secondary antibodies. Peroxidase was indicated by the bright red precipitate produced in the presence of 3-amino-9-ethyl-carbazole, and sections were counterstained with Mayer hematoxylin. For virus titrations, lungs from 5 mice were weighed, homogenized in 1 mL PBS, and clarified. The supernatants were used for virus titration by plaque or median tissue culture infectious dose assays. Because the appearance of a biphasic expiratory pattern has been shown to announce death within ≈24 h (*6*), this qualitative sign was chosen, for humane reasons, as the endpoint of the experimental disease. On this endpoint day, lungs from 5 mice were sampled and weighed, and homogenates thereof were desiccated for dry weight determination.

## Results

### Clinical, Gross Pathologic, and Virologic Observations

**Figure 1:**
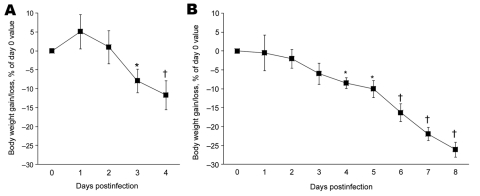
Effect of influenza A virus subtype H5N1 (A) and H1N1 (B) strains on bodyweight gain or loss after intranasal inoculation of 10× the 50% mouse lethal dose on day 0. Relative values are given, as calculated with respect to preinoculation control values (mean ± SD). For each virus strain, means significantly different from baseline are indicated (Student *t* test for paired values). *p<0.05; †p<0.01.

**Figure 2:**
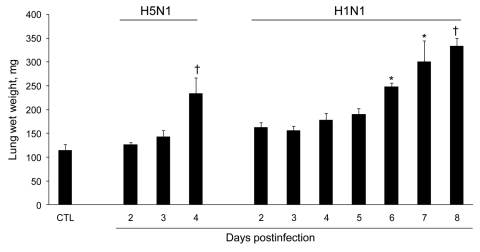
Effect of influenza A virus subtype H5N1 and H1N1 strains on lung weight after intranasal inoculation of 10× the 50% mouse lethal dose on day 0. Absolute values are given as means ± SD for 5 mice at each time point. For each virus strain, means significantly different from those of control (CTL) lungs are indicated (nonparametric Mann-Whitney test). *p<0.05; †p<0.01.

**Figure 3:**
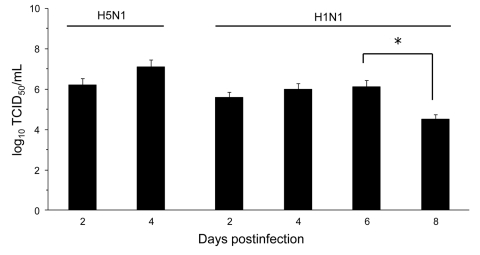
Effect of influenza A virus subtype strains H5N1 and H1N1 on lung virus titers 2–8 days after intranasal inoculation of 10× the 50% mouse lethal dose on day 0. Titers are expressed as the log_10_ median tissue culture infectious dose (TCID_50_) units per milliliter of lung homogenate. Significantly different titers are indicated (nonparametric Mann-Whitney test). Error bars indicate SD calculated from individual virus titers. *p <0.05.

The influenza A virus strains (subtypes H1N1 and H5N1) used in this study were isolated, respectively, from a diseased pig in the United States in 1976 and from a crested eagle smuggled from Thailand in 2003 (*7*). Both were nonpathogenic for FVB/J mice (MLD_50_ >10^6^ PFU/50% tissue culture infective dose [TCID_50_]). After adaptation, the strains showed a similar pathogenic outcome in FVB/J mice, i.e., close MLD_50_ values: 3.2 PFUs for the subtype H1N1 strain and 6.4 TCID_50_ for the subtype H5N1 strain. These results allowed a relevant comparison of their respective pathologic signatures. Overall, virus-associated illness, bodyweight loss, and gross lesions caused by inoculation of 10 MLD_50_ were similar for both viruses, except that body condition and respiratory function deteriorated far more rapidly after subtype H5N1 inoculation, the endpoint being reached on 4 dpi for subtype H5N1–induced disease and 8 dpi for subtype H1N1–induced disease. The pathologic processes caused no symptoms for the first 2 (H5N1) or 3 (H1N1) days and then gave rise to general signs such as gradually slower, less frequent, and more erratic spontaneous displacements and a ruffled coat. By 3 dpi (H5N1) or 5 dpi (H1N1), all mice became lethargic and abruptly showed clinical signs of respiratory disease, including respiratory distress, labored breathing, and forced expiration. Mice inoculated with subtype H5N1 lost 10% of their bodyweight during the last 48 hours before the endpoint day. In mice that were inoculated with subtype H1N1, weight loss was acute and biphasic: a 10% loss occurred between virus inoculation and the appearance of respiratory symptoms, and an additional 20% was lost during ARDS (Figure 1). Autopsies performed on the endpoint day of subtype H1N1 disease consistently showed dark, purplish, bulky, noncrepitant, liverlike lungs, findings compatible with a diagnosis of massive pulmonary congestion and consolidation. In subtype H5N1–inoculated mice, the lungs at endpoint were bulky, noncrepitant, and diffusely pinkish gray, which suggests a diagnosis of congestion with massive pulmonary edema. Mice inoculated with either virus had a lung wet weight at endpoint approximately double that of controls, but this weight gain was achieved during the last ≈24 hours in mice inoculated with subtype H5N1, whereas mice inoculated with subtype H1N1 showed a progressive lung weight increase over 96 hours, from 4 dpi to the endpoint day (Figure 2). At the endpoint, the dry/wet weight ratio of the lungs was ≈22% lower for subtype H5N1-infected mice (17.6% ± 1.1%) than for subtype H1N1-infected mice (21.4% ± 1.4%). No obvious gross lesions were observed in the heart, liver, spleen, kidney, brain, or perivisceral fat. The lung virus loads measured on 2, 4, 6, and 8 dpi are shown in Figure 3. The time required to reach the peak virus titer was the same for both virus strains. Death occurred at the peak lung virus concentration for subtype H5N1, but subtype H1N1–associated disease did not become fatal until 4 days after this peak, when virus clearance was already substantial (Figure 3).

### Histopathologic Observations

**Figure 4:**
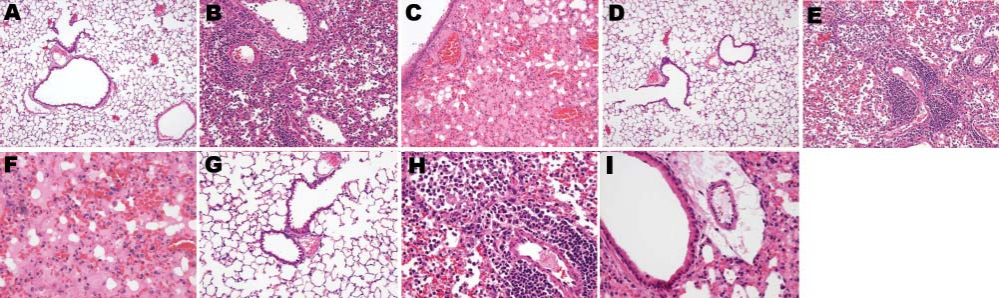
Photomicrographs of the lung sections of influenza A virus (H1N1)– and (H5N1)–infected mice at endpoint (hematoxylin and eosin stain). Dramatically different histopathologic signatures are observed, with either a mostly cellular reaction (H1N1) or a mostly humoral reaction (H5N1). Panels A, D, and G: 3 views of vehicle-infected lungs (original magnification ×100). Panels B and E, subtype H1N1: Dense granulocytic and lymphocytic cell infiltrates in the interstitium and around vessels and airways with focally denuded lamina propria due to epithelial necrosis and desquamation (original magnification ×100). Panel C, subtype H5N1: Airway epithelium is intact; note the striking difference in the number of infiltrated inflammatory cells between subtypes H1N1- and H5N1-infected lungs. Dramatic congestion of the vessels is visible, with extensive interstitial and alveolar edema (original magnification ×100). Panel F, subtype H5N1: Alveoli are completely filled with edema and hemorrhages; cellular infiltrates are conspicuously absent (original magnification ×200). Panel H, subtype H1N1: An airway with a totally denuded lamina propria is shown (top, left), with its lumen filled with granulocytic and lymphocytic exsudate (original magnification ×200). A prominent periarteriolar lymphocytic cuff is visible (bottom right). Panel I, subtype H5N1: Moderate inflammatory cell infiltrate, with no cuffing of any airway or vessel; an airway with a still intact epithelium is shown, located just beside a vessel with dramatic peripheral edema (original magnification ×200).

**Figure 5:**
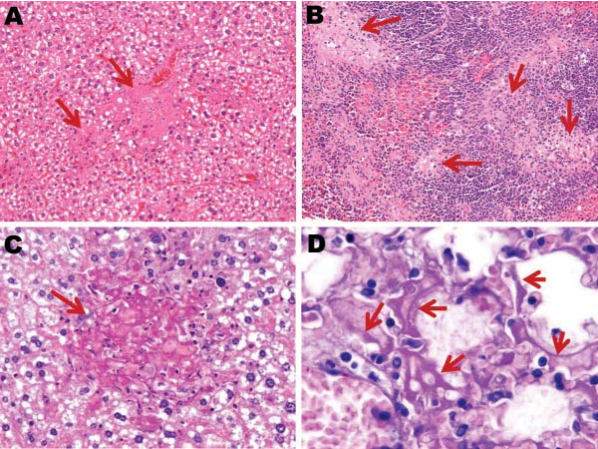
Photomicrographs of liver, spleen, and lung sections from influenza virus A (H5N1)–infected mice at endpoint. Necrotic foci (arrows) scattered throughout the liver (A) (original magnification ×200) and spleen (B) (original magnification ×100) from subtype H5N1–infected mice (hematoxylin and eosin stain); these foci are absent from subtype H1N1–infected mouse livers. C) Necrotic foci in the liver stain periodic acid–Schiff (PAS)–positive (arrow), which suggests focal accumulation of glycogen (original magnification ×400). D) Numerous alveolar walls lined with PAS-stained hyaline membranes (arrows), suggestive of necrosis and desquamation of pneumocytes (original magnification ×1,000).

An exhaustive list of the histopathologic lesions caused by the 2 viruses is given in Table A1. Some changes in lung morphology were identical for both viruses. First, a clear topographic extension of the lesions was perceptible between the first and the last day of infection, with centrifugal spreading from the terminal bronchioles or the alveoli adjacent to the airways. Qualitatively, all alterations characterizing the exudative phase of the histopathologic condition termed diffuse alveolar damage were identifiable, with intense congestion of the alveolar capillaries, marginated intracapillary neutrophils, necrosis of the alveolar epithelium, interstitial and alveolar edema, hyaline membranes, and invasion of the alveoli by (mostly) mononucleate cells. On the other hand, we did not observe cuboidalization of the alveoli (hyperplasia of type II pneumocytes) or hyperplasia or squamous metaplasia of the airway epithelia. These results indicate extremely rapid disease progression, nearly complete elimination of type II pneumocytes, or both. Despite these similarities, when sections of lung tissue samples taken on the last day from infected mice were pooled by subtype, an examiner unaware of which infection he was looking could easily distinguish one from the other (Figures 4, 5). The criteria for attributing lung lesions to the subtype H1N1 strain were the following: 1) earlier and much more extensive degeneration, necrosis, and desquamation of the airway epithelium; 2) a much higher cell density of the peribronchial, peribronchiolar, interstitial, and intra-alveolar infiltrates; 3) the presence of dense cuffs of mononucleate cells around the arterioles; 4) far less extensive alveolar edemas; and 5) the rarity of alveolar hemorrhages. The lesions caused by the subtype H5N1 strain were distinguishable by the late and mild regressive alterations of the airway epithelium, the extent of alveolar edema, the low cell density of the inflammatory infiltrates, the high number of alveolar hemorrhage foci, and the unusual appearance of the pulmonary arterioles (which seemed to have been dissected from the surrounding tissues because of the magnitude of the perivascular edema).

On the other hand, no arteriole showed any cuff of infiltrated mononucleate cells. Some blood-vessel walls also showed hemorrhage inside the muscle layer. No other organ examined was found to carry any histopathologic lesions except, notably, the liver in subtype H5N1–infected mice (Figure 5). These livers displayed multifocal necrosis, with necrotic foci consisting of aggregates of hypereosinophilic, pyknotic, and caryorhectic hepatocytes, admixed with a few neutrophils and lymphocytes. Such foci were also seen in the spleen in some animals. Strikingly, numerous PAS-positive islets were detected throughout the livers of subtype H5N1–infected animals, each overlapping with a necrotic focus. Patterns of centrolobular, hydropic, granular (2 dpi), centrolobular (3 dpi), and panlobular (4 dpi) microvesicular fatty degeneration were also observed in the livers of all subtype H5N1-infected animals. Interstitial hemorrhages were seen in the renal medulla.

### Detection of Viruses in Tissues

The results of immunohistochemical tests were homogeneous for mice infected with the same strain. Overall, they showed that the subtype H1N1 strain swarmed centrifugally from the bronchioles throughout the lungs over 4–5 days, but remained strictly confined to the lungs. The subtype H5N1 virus, in contrast, conquered the whole lung over 24–48 hours; infected some bronchioles only later; and spread to the liver, pancreas, kidneys, spleen, brain, and perivisceral fat.

### Topologic Distribution of Subtype H1N1 Antigens over Time

**Figure 6:**
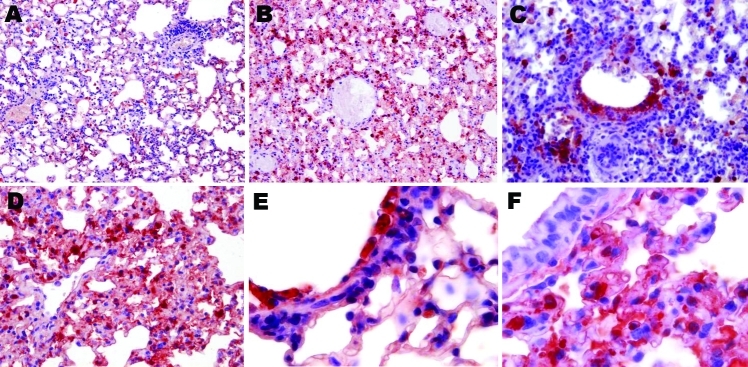
Topologic distribution of influenza antigens in the lungs of mice infected with influenza virus A subtype H1N1 and H5N1 strains at endpoint (antinucleoprotein immunohistochemical staining). A) Subtype H1N1 and B) subtype H5N1, both showing diffusely distributed positive staining of numerous pneumocytes and alveolar macrophages (original magnification ×100). C) Subtype H1N1, showing antigens massively present in the remaining non-desquamated airway epithelial cells (original magnification ×400); viral amplification in type I and type II pneumocytes is far more intense and widespread 4 days after inoculation of the subtype H5N1 virus (D) than 7 days after inoculation of the subtype H1N1 virus (original magnification ×400). E) Desquamated, necrotic, and intensely virus-positive airway epithelial cells in a terminal bronchiole and adjacent alveoli of a mouse infected with subtype H1N1, compared with F) uninfected, intact airway epithelial cells in a terminal bronchiole and adjacent alveoli of a mouse infected with subtype H5N1, illustrating the different pneumotropism of the 2 viruses (original magnification ×1,000). Conversely, the density of virus-positive cells in the lung/alveoli is higher after inoculation of the subtype H5N1 strain (Mayer hematoxylin counterstain).

The virus was first detectable in the epithelium of the bronchi and bronchioles on 3 dpi. By 5 dpi, the stain was more conspicuous and appeared also in the alveolar epithelium of the areas adjacent to the airways. By 7 dpi, the virus was detectable in the epithelia of almost all bronchi and bronchioles and in the alveolar epithelium in extensive areas of the lungs. In the alveolar structures, staining showed the virus in type I and type II pneumocytes and in alveolar macrophages (Figure 6, panels A, C, and E). Nonrespiratory organs sampled on 3, 5, or 7 dpi remained strictly virus negative.

### Topologic Distribution of Subtype H5N1 Antigens over Time

**Figure 7:**
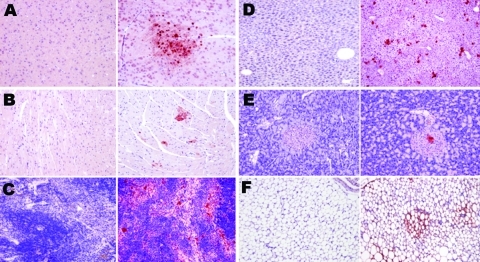
Topologic distribution of antigens in mice infected with influenza A virus subtype H1N1 at day 7 postinfection (left columns) and subtype H5N1 at day 4 postinfection (right columns) in various nonrespiratory organs. A) Glial cells (mostly oligodendrocytes); B) cardiomyocytes; C) spleen macrophages; D) hepatocytes; E) islets of Langerhans cells in the pancreas; and F) adipocytes. Bright virus-positive staining can be seen in subtype H5N1–infected mice (antinucleoprotein immunohistochemical staining), while absence of any staining can be seen in subtype H1N1–infected mice (Mayer hematoxylin counterstain). Original magnification ×100.

The virus was detectable from 2 dpi in some type II pneumocytes in peribronchiolar alveoli, some interstitial/alveolar macrophages, and some endothelial cells in the vicinity of the positive alveoli. In contrast, no nonrespiratory organ examined showed any virus-positive cells. By 3 dpi, staining of the airway epithelium was still discrete and limited, whereas the alveolar epithelium showed more pronounced staining, diffusely distributed throughout the lung. In the liver, multiple nests of positive hepatocytes were detectable, corresponding exactly with the above-mentioned necrotic PAS-positive foci. A few renal tubular epithelial cells were also positive. On 4 dpi, the alveolar epithelium was still diffusely stained, but more intensely than on 3 dpi. For the first time, staining of the bronchiolar epithelium was also visible, but not all bronchioles—far from all, in fact—showed this staining. Type II pneumocytes and alveolar macrophages were more often positive than type I pneumocytes (Figure 6, panels B, D, and F). The appearance of the kidneys and liver was the same as on 3 dpi, with more conspicuous staining. Additionally, virus-positive glial cells, splenic macrophages, cardiomyocytes, islets of Langerhans cells, and peritoneal adipocytes were also detected (Figure 7).

## Discussion

Two influenza A viruses of different subtypes, derived from different species and showing no pathogenicity toward mice, were forced to evolve by serial passaging in mouse lungs. The 2 adapted viruses obtained showed practically identical virulence levels, with similar MLD_50_ values. On the basis of this criterion, they appear to be more virulent than most other viruses used to date in murine models (*4**,**8**–**16*). Their virulence is of the same order of magnitude as those of the A/Vietnam/1203/2004 (H5N1) and A/Vietnam/1204/2004 (H5N1) viruses, whose respective MLD_50_s are 0.7 and 2.1 PFUs (*17*). In both cases, inoculation of 10 MLD_50_ causes biphasic weight loss, culminating in death with a loss of ≈10% (H5N1) or ≈25% (H1N1) bodyweight. Viral amplification is maximal for both viruses on 4 dpi, roughly corresponding to the typical inoculation-to-peak lag of natural murine respiratory viruses (*6**,**18*). On the other hand, the 2 viruses adapted in the lungs showed replication kinetics that differed substantially from what is observed with natural viruses, with a quasi-plateau from 2 to 5/6 dpi instead of the classical Gaussian profile. Notably, this peculiar amplification kinetics profile has been described previously for mice infected with mouse-adapted forms of the A/Puerto Rico/8/34 (H1N1) virus (*19*), the A/South Carolina/1/18 (H1N1) virus (*4*), and several human subtype H5N1 strains showing high or low pathogenicity (*4**,**14*). These reports suggest that this profile is typical of influenza virus amplification by the murine respiratory system.

A final common feature of infection with the 2 virus subtypes was diffuse alveolar damage, which dominates both histopathologic profiles; these results corroborate the pathologic data found in the literature. Seasonal human influenza epidemics typically consist of a transient tracheobronchitis caused by preferential attachment of the virus to the laryngeal, tracheal, and bronchial epithelia. In contrast, those influenza viruses which are highly pathogenic toward humans, from the pandemic viruses of 1918 (H1N1), 1957 (H2N2), and 1968 (H3N2) to the subtype H5N1 strains isolated from humans since 2003, additionally colonize the bronchiolar and alveolar epithelia, preferentially or not, and cause diffuse alveolar damage as an additional primary lesion (*20**–**23*). The same lesion has been found in experimental animals injected with a recent subtype H5N1 strain (*14**,**24**–**26*).

Although both viruses share the same pathogenicity, replication kinetics, and concentration peak, and although they both evoke diffuse alveolar damage by the endpoint day, they differ dramatically in terms of the ARDS course and pathologic signature. Flagrant differences make it easy to distinguish infections by the 2 subtypes. In subtype H1N1 infection, the disease becomes fatal at a point when the pulmonary edema is much less intense and leaves a histopathologic picture characterized by much more dense inflammatory cell infiltrates, generating cuffs around the bronchioles and blood vessels. Second, subtype H1N1 colonizes the epithelia of both the upper and lower airways, without any obvious preference, whereas subtype H5N1 remains confined essentially to the alveoli and terminal bronchioles. Within the alveoli, unlike the subtype H1N1 strain, the subtype H5N1 strain shows a preferential tropism for type II pneumocytes and alveolar macrophages. Lastly, whereas subtype H1N1 remains strictly confined to the respiratory system, subtype H5N1 spreads to other organs. These differences demonstrate unambiguously that the 2 highly virulent influenza A viruses studied here cause 2 different forms of ARDS. This finding suggests that the physiopathologic data obtained when studying 1 virulent strain should not be extrapolated automatically to other strains. The observed differences also suggest that diverse constellations of critical mutations in the viral genome might lead to the same fatal result.

This work addresses the question of possible differences between 2 fatal diseases caused by influenza A viruses, although some previous evidence that pointed in the same direction has already been reported. For example, the pandemic human strains of 1918, 1957, and 1968, on the one hand, and the recent subtype H5N1 strains, on the other, show different tropisms: panepithelial for the former strains (*20**,**27**,**28*) and limited to the bronchiolar and alveolar epithelia for the latter strains, a result compatible with our own observations on mouse-adapted viruses. Likewise, a panepithelial tropism has been observed for the A/South Carolina/1/18 (H1N1) virus in mice (*29*), whereas a preference for the bronchioles and alveoli has been noted for recent subtype H5N1 strains that have been injected into macaques, mice, ferrets, and cats (*14**,**25**,**30**–**37*). In addition, the observed strict confinement of our subtype H1N1 strain to the respiratory system confirms previously reported data that refute the existence of polysystemic dissemination of non-H5 viruses that are lethal to humans or laboratory animals (*20**,**27**,**29**,**38*). Conversely, our observation that the subtype H5N1 strain spreads beyond the respiratory system confirms similar observations of both humans (*22**,**39*) and laboratory animals (*14**,**24**,**25**,**30**–**34*).

Although other subtype H5N1 and subtype H1N1 viruses infect other susceptible hosts, they may not show trends similar to those observed here. These results, when integrated with the diverse pieces of evidence reported elsewhere, suggest that fatal infections caused by different highly virulent influenza A viruses do not necessarily share the same pathogenesis. To be convinced, one has only to note the ease of distinguishing, in the absence of any virus labeling, the histopathologic sections typical of the 2 strains used here (Figure 4). These different histopathologic signatures and different pathogeneses probably reflect the presence of specific sets of virulence markers that will have to be decrypted to anticipate the emergence of a pandemic. In this respect, sequence analysis of both strains will lead to insight on specific residues that are relevant for the adaptation and virulence of an influenza strain in a new host.

Furthermore, the differences between these 2 strains suggest that >1 universal cytokine storm underlies fatal influenza diseases. Thus, it might be advantageous to tailor the therapeutic approach to the influenza virus pathotype.

## Appendix

**Table A1:** Histological alterations recorded after inoculation of mice with 10 50% mouse lethal doses of 2 influenza virus strains

Tissue type	Subtype H1N1 strain				Subtype H5N1 strain		
	Day 3 pi	Day 5 pi	Day 7 pi		Day 2 pi	Day 3 pi	Day 4 pi
Bronchi and bronchioli							
Lumen	Mostly granulocytic exsudate, no blood	Granulocytic/lymphocytic exsudate, no blood	Mostly lymphocytic exsudate, no blood		Empty	Empty	Some airways contain blood
Epithelium	Partial necrosis + desquamation, all airways involved	Complete necrosis + desquamation, all airways involved	Complete necrosis + desquamation, all airways involved		Intact	Intact or only some necrotic cells	Intact or only some necrotic cells
Lamina propria	Infiltration, granulocytic, lymphocytic, macrophagic, all airways involved	Like day 3 pi, but infiltrations more severe	Like day 5 pi, infiltrated cells mostly mononuclear		Normal	Normal	Normal
Submucosa (nonalveolar interstitium)	Moderate infiltrations, lymphocytic, all airways involved, no cuffs	Like day 3 pi, but infiltrations more severe	Dense lymphocytic cuffing, all airways involved		Few interspersed surnumerary mononucleated inflammatory cells	Like day 2 pi	Like day 3 pi
Arteries							
Wall	Normal	Normal	Normal		Normal	Normal	Hemorrhages, some arteries involved
Perivascular area	Moderate lymphocytic infiltration, many arteries involved	Marked lymphocytic infiltration, many arteries involved	No edema, lymphocytic cuffs, many arteries involved		Marked edema, only scattered lymphocyte infiltration, many arteries involved	Aggravated edema, few infiltrated cells, many arteries involved	Very severe edema, few infiltrated cells, all arteries involved
Alveolar ducts, sacs, and acini							
Lumen	No edema/hemorrhage, slight lymphocytic and macrophagic infiltration, only airway-adjacent acini involved	Like day 3 pi but mostly macrophage accumulation, airway-centered foci tend to spread and coalesce	Still no edema nor hemorrhages, severe lymphocytic and macrophagic infiltrations throughout the lungs		Moderate granulocytic/lymphocytic infiltration, only airway-adjacent acini involved, no edema	Like day 2 pi but airway-centered foci already coalesce, scattered flooded alveoli	Extensive edema and hemorrhages, moderate lymphocytic infiltration
Epithelium	No necrosis seen	No necrosis seen	Some necrotic cells visible, scattered hyaline membranes		Necrosis, sometimes	Multifocal necrosis, hyalin membranes	Multifocal necrosis, numerous hyalin membranes
Interstitium	Slight lymphocytic and macrophagic infiltration, only airway-adjacent acini involved	Like day 3 pi but more congestion and airway-centered foci tend to spread and coalesce	Diffuse and marked congestion, severe lymphocytic and macrophagic infiltrations throughout the lungs		Granulocytic/ lymphocytic moderate infiltration, only airway-adjacent acini involved	Like day 2 pi but airway-centered foci already coalesce, diffuse and severe congestion,	Diffuse and severe congestion, hemorrhages and moderate lymphocytic infiltration
Nonrespiratory tissues							
Heart	No lesion seen	No lesion seen	No lesion seen		No lesion seen	No lesion seen	No lesion seen
Liver	No lesion seen	No lesion seen	No lesion seen		Centrolobular hydropic/granular degeneration	Centrolobular microvasicular fatty degeneration + multifocal granulocytic/lymphocytic necrotizing hepatitis	Panlobular microvasicular fatty degeneration + multifocal granulocytic/lymphocytic necrotizing hepatitis
Spleen	No lesion seen	No lesion seen	No lesion seen		No lesion seen	multifocal foci of necrosis	multifocal foci of necrosis
Kidney	No lesion seen	No lesion seen	No lesion seen		No lesion seen	No lesion seen	multifocal hemorrhages in medulla
Brain	No lesion seen	No lesion seen	No lesion seen		No lesion seen	No lesion seen	No lesion seen

## References

[1] Murray CJ, Lopez AD, Chin B, Feehan D, Hill KH. Estimation of potential global pandemic influenza mortality on the basis of vital registry data from the 1918–20 pandemic: a quantitative analysis. Lancet. 2006;368:2211–8. 10.1016/S0140-6736(06)69895-4 17189032

[2] Korteweg C, Gu J. Pathology, molecular biology, and pathogenesis of avian influenza A (H5N1) infection in humans. Am J Pathol. 2008;172:1155–70. 10.2353/ajpath.2008.070791 18403604 PMC2329826

[3] Kuiken T, Taubenberger JK. Pathology of human influenza revisited. Vaccine. 2008;26:D59–66. 10.1016/j.vaccine.2008.07.025 19230162 PMC2605683

[4] Perrone LA, Plowden JK, García-Sastre A, Katz JM, Tumpey TM. H5N1 and 1918 pandemic influenza virus infection results in early and excessive infiltration of macrophages and neutrophils in the lungs of mice. PLoS Pathog. 2008;4:e1000115. 10.1371/journal.ppat.1000115 18670648 PMC2483250

[5] Reed LJ, Muench HA. A simple method of estimating fifty per cent endpoints. Am J Hyg. 1938;27:493–7.

[6] Anh BD, Faisca P, Desmecht DJ. Differential resistance/susceptibility patterns to pneumovirus infection among inbred mouse strains. Am J Physiol Lung Cell Mol Physiol. 2006;291:L426–35. 10.1152/ajplung.00483.2005 16556725

[7] Van Borm S, Thomas I, Hanquet G, Lambrecht B, Boschmans M, Dupont G, Highly pathogenic H5N1 influenza virus in smuggled Thai eagles, Belgium. Emerg Infect Dis. 2005;11:702–5. 15890123 10.3201/eid1105.050211 PMC3320388

[8] Brown EG, Liu H, Kit LC, Baird S, Nesrallah M. Pattern of mutation in the genome of influenza A virus on adaptation to increased virulence in the mouse lung: identification of functional themes. Proc Natl Acad Sci U S A. 2001;98:6883–8. 10.1073/pnas.111165798 11371620 PMC34447

[9] Evseenko VA, Bukin EK, Zaykovskaya AV, Sharshov KA, Ternovoi VA, Ignatyev GM, Experimental infection of H5N1 HPAI in BALB/c mice. Virol J. 2007;4:77–82. 10.1186/1743-422X-4-77 17662125 PMC1973068

[10] Isobe H, Alt F, Bona CA, Schulman J. Intact antiinfluenza virus immune response in targeted kappa-deficient mice. Viral Immunol. 1994;7:25–30. 10.1089/vim.1994.7.25 7986333

[11] Kawaoka Y. Equine H7N7 influenza A viruses are highly pathogenic in mice without adaptation: potential use as an animal model. J Virol. 1991;65:3891–4. 2041098 10.1128/jvi.65.7.3891-3894.1991 PMC241422

[12] Lipatov AS, Andreansky S, Webby RJ, Hulse DJ, Rehg JE, Krauss S, Pathogenesis of Hong Kong H5N1 influenza virus NS gene reassortants in mice: the role of cytokines and B- and T-cell responses. J Gen Virol. 2005;86:1121–30. 10.1099/vir.0.80663-0 15784906

[13] Lu X, Tumpey TM, Morken T, Zaki SR, Cox NJ, Katz JM. A mouse model for the evaluation of pathogenesis and immunity to influenza A (H5N1) viruses isolated from humans. J Virol. 1999;73:5903–11. 10364342 10.1128/jvi.73.7.5903-5911.1999 PMC112651

[14] Maines TR, Lu XH, Erb SM, Edwards L, Guarner J, Greer PW, Avian influenza (H5N1) viruses isolated from humans in Asia in 2004 exhibit increased virulence in mammals. J Virol. 2005;79:11788–800. 10.1128/JVI.79.18.11788-11800.2005 16140756 PMC1212624

[15] Tumpey TM, García-Sastre A, Taubenberger JK, Palese P, Swayne DE, Basler CF. Pathogenicity and immunogenicity of influenza viruses with genes from the 1918 pandemic virus. Proc Natl Acad Sci U S A. 2004;101:3166–71. 10.1073/pnas.0308391100 14963236 PMC365761

[16] Tumpey TM, Basler CF, Aguilar PV, Zeng H, Solórzano A, Swayne DE, Characterization of the reconstructed 1918 Spanish influenza pandemic virus. Science. 2005;310:77–80. 10.1126/science.1119392 16210530

[17] Hatta M, Hatta Y, Kim JH, Watanabe S, Shinya K, Nguyen T, Growth of H5N1 influenza A viruses in the upper respiratory tracts of mice. PLoS Pathog. 2007;3:1374–9. 10.1371/journal.ppat.0030133 17922570 PMC2000968

[18] Faisca P, Anh DB, Desmecht DJ. Sendai virus-induced alterations in lung structure/function correlate with viral loads and reveal a wide resistance/susceptibility spectrum among mouse strains. Am J Physiol Lung Cell Mol Physiol. 2005;289:L777–87. 10.1152/ajplung.00240.2005 16006482

[19] Hennet T, Ziltener HJ, Frei K, Peterhans E. A kinetic study of immune mediators in the lungs of mice infected with influenza A virus. J Immunol. 1992;149:932–9. 1321855

[20] Winternitz MC, Wason IM, McNamara FP. The pathology of influenza. New Haven (CT): Yale University Press; 1920. p. 20–45.

[21] To KF, Chan PK, Chan KF, Lee WK, Lam WY, Wong KF, Pathology of fatal human infection associated with avian influenza A H5N1 virus. J Med Virol. 2001;63:242–6. 10.1002/1096-9071(200103)63:3<242::AID-JMV1007>3.0.CO;2-N 11170064

[22] Gu J, Xie Z, Gao Z, Liu J, Korteweg C, Ye J, H5N1 infection of the respiratory tract and beyond: a molecular pathology study. Lancet. 2007;370:1137–45. 10.1016/S0140-6736(07)61515-3 17905166 PMC7159293

[23] van Riel D, Munster VJ, de Wit E, Rimmelzwaan GF, Fouchier RA, Osterhaus AD, Human and avian influenza viruses target different cells in the lower respiratory tract of humans and other mammals. Am J Pathol. 2007;171:1215–23. 10.2353/ajpath.2007.070248 17717141 PMC1988871

[24] Maines TR, Chen LM, Matsuoka Y, Chen H, Rowe T, Ortin J, Lack of transmission of H5N1 avian-human reassortant influenza viruses in a ferret model. Proc Natl Acad Sci U S A. 2006;103:12121–6. 10.1073/pnas.0605134103 16880383 PMC1567706

[25] Nishimura H, Itamura S, Iwasaki T, Kurata T, Tashiro M. Characterization of human influenza A (H5N1) virus infection in mice: neuro-, pneumo- and adipotropic infection. J Gen Virol. 2000;81:2503–10. 10993940 10.1099/0022-1317-81-10-2503

[26] Salomon R, Franks J, Govorkova EA, Ilyushina NA, Yen HL, Hulse-Post DJ, The polymerase complex genes contribute to the high virulence of the human H5N1 influenza virus isolate A/Vietnam/1203/04. J Exp Med. 2006;203:689–97. 10.1084/jem.20051938 16533883 PMC2118237

[27] Opie EL, Blake FG, Small JC, Rivers TM. Respiratory disease. London (UK): H. Kimpton; 1921. p. 402–20.

[28] Herfs JF, Mulder J. Broad aspects of the pathology and pathogenesis of human influenza. Am Rev Respir Dis. 1961;83:84–97. 13713780 10.1164/arrd.1961.83.2P2.84

[29] Kash JC, Tumpey TM, Proll SC, Carter V, Perwitasari O, Thomas MJ, Genomic analysis of increased host immune and cell death responses induced by 1918 influenza virus. Nature. 2006;443:578–81. 17006449 10.1038/nature05181 PMC2615558

[30] Rimmelzwaan GF, van Riel D, Baars M, Bestebroer TM, van Amerongen G, Fouchier RA, Influenza A virus (H5N1) infection in cats causes systemic disease with potential novel routes of virus spread within and between hosts. Am J Pathol. 2006;168:176–83. 10.2353/ajpath.2006.050466 16400021 PMC1592682

[31] Govorkova EA, Rehg JE, Krauss S, Yen HL, Guan Y, Peiris M, Lethality to ferrets of H5N1 influenza viruses isolated from humans and poultry in 2004. J Virol. 2005;79:2191–8. 10.1128/JVI.79.4.2191-2198.2005 15681421 PMC546577

[32] Zitzow LA, Rowe T, Morken T, Shieh WJ, Zaki S, Katz JM. Pathogenesis of avian influenza A (H5N1) viruses in ferrets. J Virol. 2002;76:4420–9. 10.1128/JVI.76.9.4420-4429.2002 11932409 PMC155091

[33] Yen HL, Lipatov AS, Ilyushina NA, Govorkova EA, Franks J, Yilmaz N, Inefficient transmission of H5N1 influenza viruses in a ferret contact model. J Virol. 2007;81:6890–8. 10.1128/JVI.00170-07 17459930 PMC1933302

[34] Rimmelzwaan GF, Kuiken T, van Amerongen G, Bestebroer TM, Fouchier RA, Osterhaus AD. Pathogenesis of influenza A (H5N1) virus infection in a primate model. J Virol. 2001;75:6687–91. 10.1128/JVI.75.14.6687-6691.2001 11413336 PMC114392

[35] Xu T, Qiao J, Zhao L, Wang G, He G, Li K, Acute respiratory distress syndrome induced by avian influenza A (H5N1) virus in mice. Am J Respir Crit Care Med. 2006;174:1011–7. 10.1164/rccm.200511-1751OC 16917113

[36] Katz JM, Lu X, Frace AM, Morken T, Zaki SR, Tumpey TM. Pathogenesis of and immunity to avian influenza A H5 viruses. Biomed Pharmacother. 2000;54:178–87. 10.1016/S0753-3322(00)89024-1 10872716

[37] Kuiken T, Rimmelzwaan GF, Van Amerongen G, Osterhaus AD. Pathology of human influenza A (H5N1) virus infection in cynomolgus macaques (*Macaca fascicularis*). Vet Pathol. 2003;40:304–10. 10.1354/vp.40-3-304 12724572

[38] Lowen AC, Mubareka S, Tumpey TM, García-Sastre A, Palese P. The guinea pig as a transmission model for human influenza viruses. Proc Natl Acad Sci U S A. 2006;103:9988–92. 10.1073/pnas.0604157103 16785447 PMC1502566

[39] de Jong MD, Simmons CP, Thanh TT, Hien VM, Smith GJ, Chau TN, Fatal outcome of human influenza A (H5N1) is associated with high viral load and hypercytokinemia. Nat Med. 2006;12:1203–7. 10.1038/nm1477 16964257 PMC4333202

